# Cohort Profile: The Dutch Perined-Lifelines birth cohort

**DOI:** 10.1371/journal.pone.0225973

**Published:** 2019-12-05

**Authors:** Nastaran Salavati, Marian K. Bakker, Eline M. van der Beek, JanJaap H. M. Erwich

**Affiliations:** 1 Department of Obstetrics and Gynecology, University Medical Centre of Groningen, University of Groningen, Groningen, The Netherlands; 2 Department of Genetics, EUROCAT Registration Northern Netherlands, University Medical Centre of Groningen, University of Groningen, Groningen, The Netherlands; 3 Department of Pediatrics, University Medical Centre of Groningen, University of Groningen, Groningen, The Netherlands; 4 Danone Nutricia Research, Utrecht, The Netherlands; Universidade Federal do Rio de Janeiro, BRAZIL

## Abstract

**Background:**

Maternal nutrition status (e.g. dietary/nutrient intake) during pregnancy has been associated with pregnancy outcomes including birth weight, infant survival and metabolic health of the offspring during later life. During the past few years, maternal dietary intake, at least three months before conception, has been shown to affect pregnancy outcomes also. However, literature investigating this link is still scarce. The studies that have looked at preconception dietary intake in relation to pregnancy outcome were either animal studies, had small sample sizes or focused on only selected macronutrient intake rather than complete (macro)nutrient composition or dietary intakes (e.g. food groups). Therefore, we aim to investigate the association between preconception diet and pregnancy outcomes in a linked birth cohort. The main objective of this manuscript is to describe the methodology of establishing this birth cohort and to describe both the characteristics of the study population included as well as the representativeness in terms of dietary intake.

**Methods:**

We created the birth cohort by linking two existing databases; a large population-based cohort study in the Netherlands (The Lifelines Cohort study) and the Dutch national birth registry (Perined), through a ‘trusted third party’. The birth cohort contains information on maternal dietary intake during preconception as well as pregnancy outcomes.

**Results and discussion:**

In the Lifelines Cohort study, 3,418 pregnancies were available for linking with Perined. In total, 2,368 pregnancies (86.9%) were linked with Perined, resulting in the birth cohort. With this linked cohort we are able to provide insights on the associations between dietary intake before conception and pregnancy outcomes. Such data could potentially improve nutritional care for women of childbearing age. Lifestyle changes in the period preceding pregnancy may be most effective in improving pregnancy outcomes. A focus on this window of opportunity may provide both sufficient time, as well as a period when women are potentially motivated to adopt health optimizing behaviours.

## Introduction

The central role of nutrition and metabolism in pregnancy for health and well-being of pregnant women, pregnancy outcomes, and long-term health and development of the offspring has been generally recognized. Maternal nutrition during pregnancy has been linked to birth outcomes including fetal growth, gestation length, congenital anomalies, as well as long term health effects for the offspring through intra-uterine programming with impact on later susceptibility to for instance cardiovascular diseases and Type 2 Diabetes Mellitus [1,2]. Congenital anomalies, preterm birth and low birth weight are some of the adverse pregnancy outcomes that were influenced by dietary deficiencies, and these contribute greatly to neonatal mortality and the global burden of disability later in life [3,4]. There is increasing recognition that also a woman’s nutritional status before pregnancy affects maternal and child outcomes. Animal studies suggested that diet may influence oocyte quality during the preconception period as well as placenta and early embryonic development during the first trimester of pregnancy, and thereby the proficiency of the fetus, fetal development and finally health throughout life [5]. Formation of most organs and important physiological changes in the mother occurs mostly between the third and seventh week after the last menstrual period, when many women are not yet aware of being pregnant. Consequently, several teratogenic effects have been associated with dietary and/or environmental exposures during this time [6]. Therefore, it is important to optimize the woman’s dietary intake in the periconceptional period to ensure optimal outcomes. De-Regil et al. [7] discussed the current guidelines on preconceptional nutrition interventions for girls and women and highlighted the gaps in evidence and guidelines. They found that, with the exception of vitamin A, folic acid, iodine and a few other nutrients [8], literature about the link between nutritional status before conception and birth outcomes was scarce. Therefore, further research needs to be done to study possible associations between preconceptional diet, fetal growth and pregnancy outcomes in large cohorts. Although there are already some existing birth cohorts (e.g. Southampton Womens Study [9]), most research in this area was focused on nutrition and lifestyle factors during (early) pregnancy, rather than the preconception period. In this manuscript we describe how we have used available dietary data from a large population-based cohort study in the Northern Netherlands (Lifelines) and linked it to the available data in the Dutch Perinatal Registry (Perined) to create a Perined-Lifelines linked birth cohort that can be used to investigate the possible association between dietary intake in the preconception period and pregnancy outcomes. In this paper, we will describe the characteristics of the Perined-Lifelines linked birth cohort and investigate to what extent this cohort is a representative sample of the Northern Netherlands population in terms of dietary intake.

## Materials and methods

The Perined-Lifelines linked birth cohort consisted of female participants of childbearing age participating in the Lifelines Cohort Study, who became pregnant during their enrollment in Lifelines. Since information about the course of the pregnancy and pregnancy outcomes were not routinely collected in Lifelines, information on these pregnancies and pregnancy outcomes was derived from the Dutch Perinatal Registry (Perined).

### Overall design Lifelines Cohort Study

The Lifelines Cohort Study is a prospective population-based cohort study examining the health and health-related behaviors of 167,729 persons living in the Northern Netherlands. The overall design and rationale of the study has been described in detail elsewhere [10]. In brief, between 2006 and 2013, inhabitants aged between 25 and 50 living in the northern part of the Netherlands were invited by their general practitioners (GP) to participate in Lifelines. Individuals with severe psychiatric or physical illness, limited life expectancy (<5 years) or insufficient knowledge of the Dutch language to complete a Dutch questionnaire were not considered eligible by the GP and not invited to participate. In addition, individuals who were not invited by their GP and did not meet above mentioned excluding criteria, were able to enroll themselves in the study via the Lifelines website.

Individuals who were interested received an informed consent form. After this informed consent was signed, the participants received a baseline questionnaire and an invitation for a health assessment at the Lifelines research site. During this visit, participants were asked whether family members would also be willing to participate, thereby contributing to the three-generation design. Overall, 49% of the participants (n = 81,652) were invited through their GP, 38% (n = 64,489) via participating family members and 13% (n = 21,588) self-registered via the Lifelines website. It was shown that the Lifelines population can be generalized to the general population and recruitment strategy was not subject to selection bias as it had minor effect on the level of representativeness [11].

#### Data collection

Information on lifestyle factors and diet was collected at enrollment (baseline), during follow up (e.g. questionnaires), and during visits at the Lifelines research locations. The time between the baseline questionnaires and the first follow-up questionnaire was approximately two years. The time between the first follow-up questionnaire and the second follow-up questionnaire was also approximately two years. A comprehensive overview of the data collection can be found at the Lifelines catalogue at www.lifelines.nl. Self-administered questionnaires were used to collect data regarding demographics (ethnicity, education) and lifestyle (smoking, alcohol, diet). Height and body weight without shoes and heavy clothing were measured during the visit to one of the Lifelines research sites, with the SECA 222 stadiometer and the SECA 761 scale. Body mass index (BMI) in kg/m^2^ was calculated. Dietary intake was retrieved through a 110 item semi-quantitative baseline food frequency questionnaire (FFQ) assessing food intake over the previous month. This FFQ was developed by Wageningen University using the Dutch FFQTOOL^TM^, in which food items were selected based on the Dutch National Food Consumption Survey of 1997/1998 [12–14]. This FFQ was designed to include food groups that account for at least 80% of the variance and 80% of the population intake of both energy and macronutrients. Seven response categories were used to assess consumption frequency, ranging from “not this month” to “6–7 days a week”. Portion size was estimated by fixed portion sizes (e.g., slices of bread, pieces of fruit) and commonly used household measures (e.g., cups, spoons). The FFQ focused on estimates of energy and macronutrients intake and food groups, including alcohol intake, using the Dutch food composition database of 2011 [15]. Reliability of reported dietary intake was assessed by the Goldberg cut-off method, which relies on the ratio of reported energy intake and basal metabolic rate [16], calculated with the Schofield equation [17]. Dietary information was available for 144,095 adults.

### Design of Perinatal Registry of the Netherlands (Perined)

Information on pregnancy and pregnancy outcome (e.g. birth weight, gestational age, apgar score) was derived from Perined [18]. Perined is a national registry that contains data on pregnancy, obstetric history and pregnancy outcomes of more than 97% of all pregnancies in the Netherlands [19]. These data were routinely collected by 94% of midwives, 99% of gynaecologists and 68% of pediatricians including 100% of Neonatal Intensive Care Unit paediatricians [19]. Perined aims to improve the quality of perinatal care through providing data for research and on audits on adverse outcomes.

### Linking procedure: Involved parties and agreements

Within the Lifelines Cohort study, participant data has gone through a data verification procedure, the research data were pseudonomised and separated from personal data before it was made available for researchers [10]. Also the registry of Perined is anonymous, and does not contain a unique patient identifier available for researchers. Therefore, the available personal linking variables from both Perined and Lifelines, which were birth date and 4-digits ZIP code of the residential address of the female participants from Lifelines, and birth date of their child, were not available to the researchers.

Linking between the selection of female participants from Lifelines with Perined, was therefore done anonymously using a pseudonym with the assistance of a ‘trusted third party’ (ZorgTTP, Houten, The Netherlands), facilitated by Mondriaan project (UMCG)/Lygature (Utrecht, The Netherlands). ‘Mondriaan’ operated as a data-broker or intermediate bringing parties together offering and seeking health data for scientific research. Before the linking procedure was initiated, all the involved parties formulated and signed agreements to cover rights and obligations concerning handling and storing of the data (S1 Fig). ‘ZorgTTP‘ was responsible for the pseudonymisation of the data, a process that included three different phases.

First, an unique pseudonym for each female participant was made, using the three personal linking variables. If one of these three variables was missing (in either Lifelines or Perined), data from the respective female participant could not be linked to Perined data. In this phase, the ‘Privacy and Send Module’ was used at the locations of the sources (Lifelines and Perined (performed by Research Data Support, UMCG)). The available database, at both source locations, was validated, and ‘pre-pseudonyms’ were created of the personal linking variables through hashing. Personal data were removed, and the remaining dataset was divided in a part with the ‘pre-pseudonyms’ and a part with the substantive information like variables on nutrition and pregnancy at each source location,- i.e. four datasets in total. These datasets were encrypted and send to ‘ZorgTTP’ using a protected connection (HyperText Transfer Protocol Secure (HTTPS)-connection).

Within the second phase, the editing phase, ‘ZorgTTP’ decrypted the part with the ‘pre-pseudonyms’ with a private key. The ‘pre-pseudonyms’ were subsequently encrypted for a second time. ‘ZorgTTP’ had no access to the part of the data with the substantive information (e.g. nutritional, pregnancy related), which was secured and could only be decrypted by the researcher.

In the last phase, the ‘Aim- and Receive Module’ was used, whereby the receiver (the researcher) was able to download the pseudonomised data from the server of ‘ZorgTTP’. The datasets, both with the pseudonyms and with the substantive information, were decrypted by the researcher through a private key and merged subsequently. Through the corresponding pseudonyms in both Lifelines and Perined, the datasets were linked to each other, resulting a Perined-Lifelines linked birth cohort.

### Design Perined-Lifelines linked birth cohort

**Fig 1:**
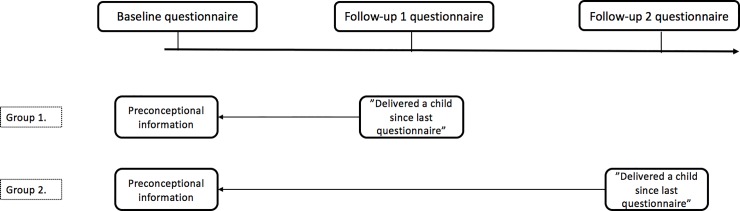
Overview Lifelines participants.

For the Perined-Lifelines linked birth cohort, we selected female participants from the Lifelines Cohort study who indicated in their first or second follow-up questionnaire to have delivered a child since the previous questionnaire. The information collected at baseline (e.g. nutrient intake through a Food Frequency Questionnaire (FFQ)) was considered as the preconceptional information available for that specific pregnancy (Fig 1). The date of birth of the child was retrieved from the Dutch population register.

#### Inclusion and exclusion criteria

Female participants who indicated to have delivered more than one child in the period between two consecutive questionnaires were excluded for further analysis, as it would be difficult to link the correct date of birth from the Dutch population register to the corresponding child, and could result in a less reliable linked dataset. After linkage, we excluded pregnancies with preterm births (gestational age <37 weeks) and women with missing or unreliable reported dietary intake. The reliability of reported dietary intake was checked using the Goldberg cut-off method, which relies on the ratio of reported energy intake and basal metabolic rate [16] calculated with the Schofield equation [17]. The reported dietary intake of women with a ratio below 0.87 or above 2.75 was considered not reliable. In addition, intake by the women less than 500 kcal/day was considered as implausible energy intake, and therefore these women were excluded for further analyses [20,21].

### Dietary intake

For the women in the final linked cohort, detailed data on dietary intake was available. This included information on intake of macronutrients (carbohydrates, proteins (total, animal, plant), fat) and energy (kcal, KJ) in grams per day. In addition, the 110 FFQ items were categorized into 22 food groups (S1 Table) [22]. Every woman contained information on the intake of every specific food group in grams per day, as well as data on protein (animal and plant), fat, carbohydrate intake contribution of the different food groups.

### Ethical approval

The Lifelines Cohort Study was conducted according to the principles of the Declaration of Helsinki and is in accordance with research code University Medical Center Groningen. Within the informed consent from The Lifelines Cohort study, participants approved that their data were used for research purposes, including for linking with other existing databases. The linking procedure for the Perined-Lifelines linked birth cohort was approved as a non WMO study by the medical ethical committee of the University Medical Center Groningen, The Netherlands (METc number: 2018/506) and waived the requirement for additional informed consent. All data in this study was fully anonymized before we accessed them.

## Results

### Characteristics of the Perined-Lifelines linked birth cohort

**Fig 2:**
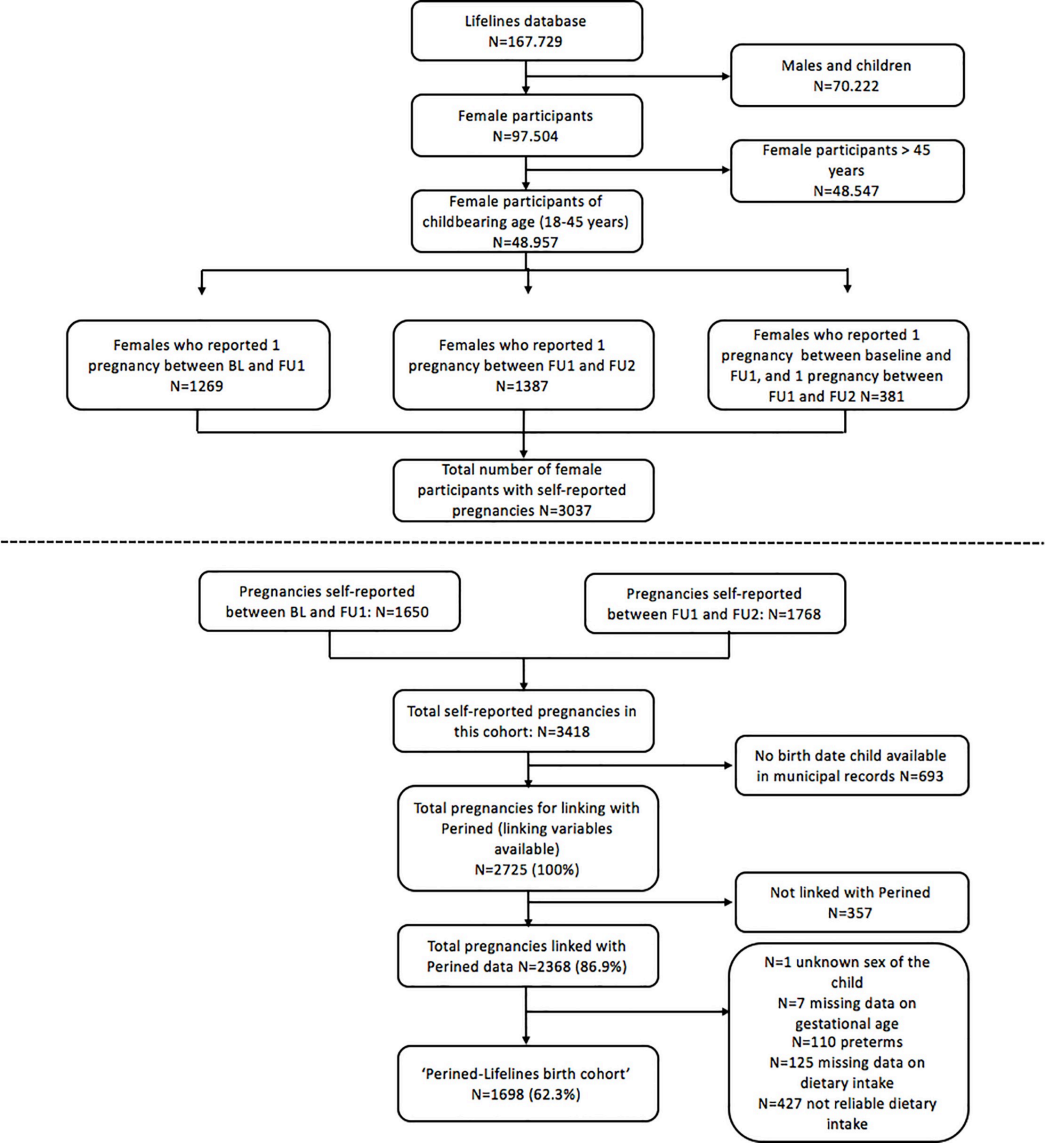
Flow chart of the Perined-Lifelines linked birth cohort.

In the period between 2006 up to the end of 2013, Lifelines included 167,729 participants, consisting of women, men and children (Fig 2). The total group of women in Lifelines was 97,504 (58.1% of which 48,957 are women of childbearing age (18–45 years, age at inclusion). Of these women 1,269 reported to have delivered a child between baseline and follow up-1, and 1,387 women reported to delivered a child between follow up-1 and follow up-2. In total, 381 women reported that they had one pregnancy between baseline and follow up-1, and again a pregnancy between follow up-1 and follow up-2. In total, there were 3,037 women with self-reported pregnancies between baseline and follow up-2 contributing to a total of 3,418 pregnancies.

The ZIP code of residential address and birth date of the women was available in Lifelines for all women. For 2,725 pregnancies a birth date of the child was retrieved in the Dutch population register and were therefore available for linking with Perined. In total, 2,368 pregnancies (86.9%) were linked with Perined. Of these women, 28 had two pregnancies in the linked cohort, one pregnancy between baseline and follow up-1 and one pregnancy between follow up-1 and follow-up 2. After linkage, we excluded pregnancies with pre-term births (gestational age <37 weeks; n = 110) and unknown sex of the child (n = 1). In addition, participants with unreliable or missing reported dietary intake were excluded from analyses (resp. n = 427 and n = 168), leaving 1,698 (62.3%) women in the birth cohort (Fig 2). Women with reliable dietary intake were more often higher educated, were younger at preconception, had a higher percentage of alcohol consumers, and lower percentage of smokers (S2 Table). They were more often nulliparous, and the median birth weight in this group was slightly lower compared to the excluded group with unreliable dietary intake (S2 Table).

As described, the resulting birth cohort contained data related to 1698 term pregnancies. The average maternal age at enrollment was 29 years (25^th^-75^th^ percentile: 27–32 years) (Table 1). Children were on average born at 39.4 weeks of gestational age with an average birth weight of 3570 grams (25^th^-75^th^ percentile: 3249–3880 grams). The majority of the children had an Apgar score of 10 at 5 min. Almost all women were of white (east/west European) ethnicity (97.8%), and the majority completed higher education (55.8%). The average maternal BMI was 23.8 kg/m^2^ (25^th^-75^th^ percentile: 21.7–26.6), and average energy intake amounted to 1813 kcal/day (25^th^-75^th^ percentile: 1545–2141). The average time between de FFQ (baseline) and the delivery of the child was 13 months (25^th^-75^th^ percentile: 11–16 months).

**Table 1:** Characteristics of the Perined-Lifelines linked birth cohort (N = 1698).

	Total cohort (n = 1698, 100%)	
**Demographics**		
Age at enrollment (years)	29	(27–32)
Ethnicity		
*White*, *East/West European Ethnicity*	1661	(97.8)
*Other*	37	(2.2)
Education^a^		
*Low*	105	(6.3)
*Moderate*	637	(37.9)
*High*	936	(55.8)
Missing	20	
Urbanization level by category ^b^		
*1*	403	(24.2)
*2*	133	(8.0)
*3*	118	(7.1)
*4*	200	(12.0)
*5*	812	(48.7)
Missing	32	
**Diet**		
Energy intake (kcal/day)	1813	(1545–2141)
Percentage energy from: ^c^		
*Carbohydrates*	46.8	(37.4–471.1)
*Mono and Di saccharides*	24.8	(22.4–284.3)
*Polysaccharides*	29.3	(21.3–292.9)
*Protein*	14.8	(15.3–128.3)
*Animal protein*	8.6	(4.0–84.7)
*Plant protein*	*6*.*0*	*(2*.*9–65*.*8)*
*Fat*	26.9	(21.1–304.8)
**Lifestyle**		
BMI ^d^ (kg/m^2^)	23.8	(21.7–26.6)
BMI WHO classification		
*<18*.*5*	19	(1.1)
*18*.*5-<25*	1019	(60.0)
*25-<30*	469	(27.6)
*≥ 30*	191	(11.3)
Alcohol		
*User percentage (%)*	1317	(77.6)
*Median consumption*^e^(g/day)	2.7	(1.4–6.4)
*Missing*	2	
Smoker	212	(12.5)
*Missing*	3	
**Pregnancy**		
Maximum time between baseline questionnaire and birth child (in months)	13.0	(11.0–16.0)
Sex of the child		
*Male*	849	(50.0)
Gravidity		
*1*	692	(40.8)
*2*	578	(34.0)
*3*	275	(16.2)
*≥4*	153	(9.0)
Parity		
*0*	813	(47.9)
*1*	632	(37.2)
*≥2*	253	(14.9)
Birth weight (in grams)	3570	(3249–3880)
*Missing*	4	
Gestational age (in weeks)	39	(39–40)
Apgar-score (after 5 min)		
*<10*	422	(24.9)
*10*	1274	(75.1)
*Missing*	2	

Data are median (IQR) or n (%). Data were complete when there is no missing row presented.

^a^Low education: primary school, vocational and lower general secondary education; Moderate education: higher secondary education and intermediate vocational training; High education: higher vocational education and university education.

^b^Level of urbanization: 1. Very high > = 2500 addresses per km^2^; 2: high 1500–2500 addresses per km^2^; 3: moderate 1000–1500 addresses per km^2^; 4: low 500–1000 addresses per km^2^; 5: rural <500 addresses per km^2^.

^c^Energy from carbohydrates, protein and fat, relative to the sum of energy from the three macronutrients.

^d^BMI = Body mass index.

^e^Median + IQR among alcohol users. One standard drink contains 10 g alcohol.

### Representativeness of dietary intake of the Perined-Lifelines linked birth cohort

To assess to what extent our study population was a representative selection of the population in terms of dietary intake we first compared intake of energy and macronutrients with the recommended reference values from the Dutch Dietary Reference Intakes (DRI) (Table 2) [23]. ‘Dietary reference intakes’ is a collective term of reference values that are quantitative estimates of nutrient intakes to be used for assessing diets of healthy people, including ‘estimated average requirement’ (EAR), ‘recommended dietary allowance’ (RDA), ‘adequate intake’ (AI) and tolerable ‘upper intake level’ (UL). Given a requirement with a normal distribution, the EAR was the level of nutrient intake estimated to meet the requirement of half of the healthy individuals in a group. This EAR was given for energy intake (in kcal/day) in Table 2. As shown, almost all female participants (n = 1695, 85.5%) consumed lower energy intake than that provided by the Dutch DRI (EAR: 2318–2437) (Table 2). The number of women with an intake below the DRI were interpreted with caution, since, as mentioned, the EAR is the level of intake that is adequate for only half of the population [23]. In addition, the FFQ was designed to include food groups that account for at least 80% of the variance and 80% of the population intake of both energy and macronutrients. Therefore, the actual energy intake of the women could be higher than that described in the FFQ results, making a strict comparison with the DRI difficult. The RDA was calculated as the EAR plus twice the standard deviation of the requirement. This described the average daily intake level that is sufficient to meet the nutrient requirement of nearly all individuals in a group. In case the RDA cannot be determined, the AI was used, a value based on observed or experimentally determined approximations of nutrient intake by a group of healthy people. This reference value was used for protein, carbohydrates and fat intake. Finally, the UL was the highest level of daily nutrient intake that is likely to elicit no risk of adverse health effects to almost all individuals in a group. In case the intake increased above the UL the risk of adverse health effects was increased. This level was known for protein and fat intake, but not for carbohydrates. This resulted in a relative narrow DRI for carbohydrates (40–42 en% AI), resulting in 83.5% of the women consuming more carbohydrates than this ‘adequate intake’ in our assessment. Intake of total protein and fat was adequate according to the guideline for majority of the female participants, respectively 99.8% and 86.6%.

**Table 2:** Number of female participants meeting dietary reference intake for macronutrients (protein, carbohydrates and fat) and energy.

	*DRI* ^*a*^	Intake below DRI		Intake according to DRI		Intake above DRI	
		Mean (SD)	N (%)	Mean (SD)	N (%)	Mean (SD)	N (%)
Energy (kcal/day)	*2318–2437 (EAR* ^*b*^*)*	1727 (328.5)	1452 (85.5)	2374 (32.6)	72 (4.3)	2737 (287.0)	174 (10.2)
Protein (En%)	*9–25 (AI* ^*c*^*-UL* ^*d*^*)*	8.8 (0.07)	3 (0.2)	14 (2.1)	1695 (99.8)	-	-
Carbohydrates (En%)	*40–42 (AI)*	37.5 (2.9)	145 (8.5)	41.2 (0.6)	136 (8.0)	48.1 (3.8)	1417 (83.5)
Fat (En%)	*20–40 (AI-UL)*	19.0 (0.8)	2 (0.1)	33.8 (3.7)	1471 (86.6)	42.4 (2.2)	225 (13.3)

^a^DRI = Dutch dietary Reference Intakes; reference intakes for Dutch non-pregnant women between 22–50 years of age [23]

^b^EAR = Estimated Average Requirements; an estimated of the average requirements of energy or nutrient needed by a group of people.

^c^AI = Adequate Intake.

^d^UL = Upper Limit.

Since our group of female participants was a selection of the Lifelines cohort participants, we also wanted to assess whether the diet quality scores from the linked Lifelines-Perined birth cohort were comparable to the complete Lifelines cohort. A recent paper assessed diet quality in the complete Lifelines cohort (adult males and females) using a Lifelines Diet Score (LLDS) [22] based on the 110 FFQ food items. The LLDS was formulated to represent relative diet quality. To account for differences in energy intake between individuals, intake of food groups was expressed in grams per 1000 kilocalories (kcal) instead of grams per day. For each food group, intake was divided into quintiles to score individual consumption relative to the complete study population (n = 129.369). The quintiles ranged from 0 to 4, with 4 points being awarded to the highest quintile of consumption for positive food groups, and to the lowest quintile of consumption for negative food groups. The sum of the 12 component scores resulted in a LLDS score, of which LLDS quintiles were made. With the absolute cutoffs from the LLDS quintiles formulated in the complete Lifelines cohort study, we compared our birth cohort in terms of diet quality. Indeed, in our cohort also approximately 20% of the female participants fell in every quintile with absolute cutoffs used comparable to the Lifelines Cohort.

## Discussion

Using data from the Dutch Perinatal Registry (Perined) linked to the Lifelines Cohort Study, we have created a new birth cohort consisting of women from childbearing age who delivered a child after their enrollment in Lifelines, providing information on dietary intakes during the period of preconception.

In this manuscript we have given an overview on the methodological approach and the development of the Perined-Lifelines linked birth cohort and described the characteristics of this group. Due to the detailed data collection on diet at lifestyle factors before pregnancy, the Lifelines Cohort offers a unique opportunity to increase the knowledge on the possible associations between specific habitual dietary intake data in the preconception period and pregnancy outcomes. Since information about the course of the pregnancy and pregnancy outcomes are not routinely collected in Lifelines, information on these pregnancies and pregnancy outcomes was derived from the Dutch Perinatal Registry (Perined). The current study set up allows us to focus on diet characteristics in the preconception period (defined as the period between baseline, when dietary intake was assessed, and follow-up questionnaires in which the participant indicated to have delivered a child since her enrollment in Lifelines). Linking these Lifelines data to the data from Perined, allowed us to investigate possible associations to pregnancy outcome.

Most of the published literature that examines the relationship between nutrition and pregnancy outcomes, has focused on data collection during the second and/or third trimester of pregnancy. However, the women’s nutritional status before conception, and/or during early pregnancy, when women are mostly unaware of their pregnancy, may influence conception as well as early placenta, embryonic and fetal development and consequently pregnancy outcomes. Literature on the relation between diet in the periconceptional period and pregnancy outcomes is still scarce.

During early pregnancy the formation of most organs as well as placental development takes place and is directly influenced by maternal health and nutritional status [24]. Animal studies have previously shown that both maternal undernutrition as well as overnutrition, before and during pregnancy, reduces placental-fetal blood flow and consequently impacts fetal growth. If the impact of nutritional status of the women could be optimized before pregnancy, outcomes for both mother and infant could be improved. Reproduction may motivate women to adapt health optimizing behaviours for the well-being of their unborn child [25]. However, several intervention studies during pregnancy showed that (long-term) beneficial lifestyle changes are difficult to maintain, and if effective, it results in only modest, short term changes [26,27]. Instead, the preconception period may be a more optimal period to intervene [28–30].

A few studies have investigated the association between preconceptional nutrient intake and pregnancy outcomes. Observational studies have suggested that pre- and periconceptional intake of vitamins and mineral supplements are associated with a reduced risk of preterm delivery, as well as delivering offspring with low birth weight. A recent systematic review concluded however that the overall quality of the evidence was low for most outcomes with the exception of the benefit of maternal preconception folic acid intake and the risk of neural tube defects [31]. Several cohort studies have suggested that dietary patterns up to three years before pregnancy, characterized by fruit, vegetables, legumes, nuts and fish and low intake of red and processed meat, are associated with reduced risk of gestational diabetes [32–35], hypertensive disorders of pregnancy [36,37], and preterm birth [38]. Since few people will plan a pregnancy three years in advance, this highlights the need for interventions on population level in future. In addition, in most cases the preconceptional period is most often determined in retrospect, underlying the importance of increasing awareness early in women of childbearing age.

The linked Perined-Lifelines birth cohort demonstrates the feasibility of linking (healthcare) databases through a pseudonymised linking procedure. Our method is generalizable to linkage of administrative data in other contexts where data is only given out anonymously to the researcher. Errors occurring during linkage (missed-matches and false matches) can result in substantially biased results: false-matches can bias associations towards the null and missed-matches can lead to a selection bias [39]. Our linkage was done on three personal identifiable variables; 4-digits ZIP code of the residential address, the birth date of the female participants from Lifelines and birth date of their child, making the chances of errors (in terms of missed-matches and false matches) low, and resulting in a high linkage rate of 86% when information for all variables used for linkage are complete. To facilitate such a linking procedure, strict access arrangements and secure data transfer processes were established. In addition, secure physical locations were created with restricted network and no internet access. In this linking procedure, several parties were involved (S1 Fig), and agreements had to be made and signed between all the different parties, making it a time consuming process.

A limitation of linking administrative or electronic healthcare data is the imperfect nature of data for research purpose, as often some information important and interesting for research may be missing [40]. For example, information on gestational weight gain was not available in this study. However, the large sample size and representativeness of this linked cohort offers a cost-effective alternative to traditional birth cohort studies, providing valuable information on dietary intake during preconception and pregnancy outcomes in a representative group of pregnant women from the Netherlands. It has previously been described that the complete Lifelines study population is broadly representative for the adult population of the north of the Netherlands [11]. Although selection bias is a risk in a population-based cohort study, the recruitment strategy of the Lifelines participants had no effect on the representativeness of the cohort, indicating that the risk of selection bias was low and that the results that will be found in the Lifelines cohort can be generalized to the general population. In terms of demographic variables our birth cohort was comparable with the complete Lifelines cohort. Recent literature showed that within the complete Lifelines cohort, participants were more often Dutch and that less women had a low educational attainment [22]. The overall alcohol user percentage and intake however was much lower in our cohort, compared to other studies [22], likely due to the fact that we have a cohort of only women, and that women tend to drink less and less often than men [41,42]. In addition, the women were of childbearing age and consequently might be more conscious to preserve a healthy lifestyle also anticipating a possible (future) pregnancy. Also in terms of dietary intake our cohort seemed to be representative both compared to the complete Lifelines cohort [22], as well as compared to the Dutch dietary guidelines [23]. However, as described, 85.5% of the women had an energy intake below the Estimated Average Requirement (EAR), which is possibly due to the fact that this DRI describes adequate requirements for around 50% of the women. Therefore, women consuming less energy intake than the EAR could still meet their individual daily energy requirement. In addition, the FFQ was not designed to describe 100% of the individuals energy intake, but to account for at least 80% of the variance of dietary intake and 80% of the population intake of both energy and macronutrients. Therefore it may be possible that the actual intake of energy was actually higher than described in the results from the FFQ. The reference values used for the intake of macronutrients (e.g. protein, fat) are way more robust as they apply for all individuals in a group. The fact that the intake of these nutrients was within the normal limits when compared to the DRI, shows that the dietary intake of our cohort is likely to be representative in comparison with the DRI.

Dietary intake was assessed via a FFQ, which was validated [43] and contained questions concerning dietary intake over the previous month. The advantage of recording dietary intake retrospectively, was is that the process of recording will not alter awareness of food intake and will not improve accuracy in completing the questionnaire, something which is the case in prospectively retrieved diet records [44]. Previous studies have shown that energy intake reported by FFQ showed, on average, very good agreement with actual energy intake during controlled feeding trials, while body weights were kept stable [13]. According to the literature, accurate reporting of energy intake was influenced by several factors including sex, age, educational level, BMI, psychosocial factors and lifestyle [13]. On most of these factors we have data available in our cohort, and are therefore able to investigate its possible effect and we can correct for it. Based on the Goldberg cut-off method, using reported energy intake and basal metabolic rate (14), and calculated with the Schofield equation (15), we excluded all women with reported unreliable dietary intake. We compared these women in terms of demographic variables with women having a reliable dietary intake reported (S2 Table). Women with reliable dietary intake were more often higher educated, slightly younger at preconception, the percentage of alcohol consumers was higher and percentages of the percentage of smokers was lower. We consider these differences logical and do not expect selection bias to have played a role. Therefore we do not expect this to influence our results. In addition, women with reliable data were more often nulliparous, and consequently had a slightly lower mean birth weight (3570 grams vs 3640 grams) but we do not consider this as a clinical relevant difference.

For all women, we used the FFQ data collected at baseline as their preconception’ dietary intake. The time between baseline, and the moment the woman delivered her child varied among women included in our birth cohort between a minimum of 5 and a maximum of 37 months. In general, it has been shown that diet tends to be quite stable over time [44], and that changes in dietary habits after conception tend to be modest and mostly reflect intake before conception [45].

Preterm births were excluded from our cohort as the causes of preterm birth are complex and the pathophysiology that triggers it, is largely unknown [46,47]. This made it difficult to adjust for underlying pathophysiology, potentially leading to results confounded by this pathophysiology, and therefore difficult to interpretate.

As described, we noticed a discrepancy in the number of self-reported deliveries by female participants from Lifelines (n = 3,418), and the registered children in the Dutch population register (n = 2,725) in the time between baseline and FU2. This could be explained by several reasons. First, it is plausible that female participants that have indicated at FU2 that they delivered a child since the last questionnaire (FU1), have misreported the pregnancy and that this pregnancy was already reported before at follow-up 1. Such a self-reported delivery will, unjustified, be counted twice in the Lifelines cohort, but is only recorded once in the Dutch population register. In addition, only babies born after at least 24 weeks of gestational age are registered in the Dutch population registers. Women who delivered a baby with a gestational period less than 24 weeks could report child delivery, but this child may not have been registered in the Dutch population register, although this number was probably very low. Clearly, we cannot explain discrepancy based on the Dutch population register since it has been shown that 98% of the addresses and persons in the Dutch Population register were recorded correctly [48].

## Conclusions

Using data from the national birth registry in the Netherlands, we established a large, linked cohort of female participants from Lifelines, that is representative of the population in north Netherlands and will enable us to investigate dietary intake during the period prior to conception and pregnancy outcomes. With this cohort we may provide insights on the influence of dietary intake before conception to pregnancy outcomes. Such data could inform the development of recommendations to improve nutritional care for women of childbearing age, aiming to improve health of women and future generations.

## Supporting information

S1 Fig Overview involved parties and agreements. 1. Data Processing Agreement; 2. Data transfer Agreement; 3. General Agreement; 4. Data Use and Access Agreement; 5. Data Services and Processor Agreement. UMCG = University Medical Centre of Groningen, TTP = Trusted Third Party.
(DOCX)

S1 Table Food groups. (DOCX)

S2 Table Characteristics stratified by reliability dietary intake. Data are median (IQR) or n (%). Data were complete when there is no missing row presented. ^a^Low education: primary school, vocational and lower general secondary education; Moderate education: higher secondary education and intermediate vocational training; High education: higher vocational education and university education. ^b^Level of urbanization: 1. Very high > = 2500 addresses per km^2^; 2: high 1500–2500 addresses per km^2^; 3: moderate 1000–1500 addresses per km^2^; 4: low 500–1000 addresses per km^2^; 5: rural <500 addresses per km^2^. ^c^Energy from carbohydrates, protein and fat, relative to the sum of energy from the three macronutrients. ^d^BMI = Body mass index. ^e^Median + IQR among alcohol users. One standard drink contains 10 g alcohol. ^1^ Willet-Schofield. ^2^Two sided p-value; Mann Whitney U test for continuous characteristics or Pearson Chi-Square for categorical characteristics.
(DOCX)
