# Supplementary material for: Cohort Profile: The Dutch Perined-Lifelines birth cohort
Source: PLoS One. 2019 Dec 5;14(12):e0225973. doi: 10.1371/journal.pone.0225973 (PMC6894836; doi:10.1371/journal.pone.0225973)
Supplement: S1 Fig — 1. Data Processing Agreement; 2. Data transfer Agreement; 3. General Agreement; 4. Data Use and Access Agreement; 5. Data Services and Processor Agreement. UMCG = University Medical Centre of Groningen, TTP = Trusted Third Party. (DOCX) [file pone.0225973.s001.docx]

**S1 Fig.**


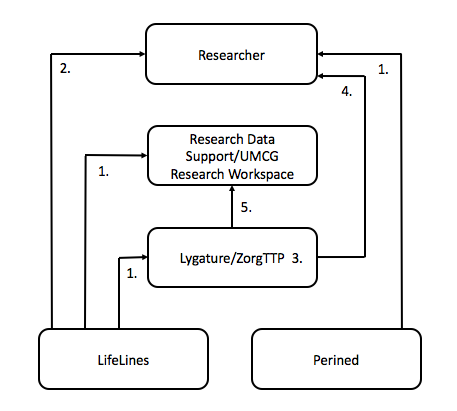


1. Data Processing Agreement; 2. Data transfer Agreement; 3. General Agreement; 4. Data Use and Access Agreement; 5. Data Services and Processor Agreement.

UMCG= University Medical Centre of Groningen, TTP= Trusted Third Party.
