# Supplementary material for: Cohort Profile: The Dutch Perined-Lifelines birth cohort
Source: PLoS One. 2019 Dec 5;14(12):e0225973. doi: 10.1371/journal.pone.0225973 (PMC6894836; doi:10.1371/journal.pone.0225973)
Supplement: S1 Table — (DOCX) [file pone.0225973.s002.docx]

**S1 Table. Food groups.**

|  | **Food groups** |
| --- | --- |
| 1 | Vegetables |
| 2 | Fruit |
| 3 | Whole grain products |
| 4 | Legumes & Nuts |
| 5 | Fish |
| 6 | Oils & soft margarines |
| 7 | Unsweetened dairy |
| 8 | Coffee |
| 9 | Tea |
| 10 | Eggs |
| 11 | Red & processed meat |
| 12 | Butter & hard margarines |
| 13 | Sugar-sweetened beverages |
| 14 | Potatoes |
| 15 | Refined grain products |
| 16 | White, unprocessed meat |
| 17 | Cheese |
| 18 | Savory & Ready products |
| 19 | Sugary products |
| 20 | Soups |
| 21 | Sweetened dairy products |
| 22 | Artificially sweetened products |
