# Supplementary material for: Cohort Profile: The Dutch Perined-Lifelines birth cohort
Source: PLoS One. 2019 Dec 5;14(12):e0225973. doi: 10.1371/journal.pone.0225973 (PMC6894836; doi:10.1371/journal.pone.0225973)
Supplement: S2 Table — Data are median (IQR) or n (%). Data were complete when there is no missing row presented. aLow education: primary school, vocational and lower general secondary education; Moderate education: higher secondary education and intermediate vocational training; High education: higher vocational education and university education. bLevel of urbanization: 1. Very high > = 2500 addresses per km2; 2: high 1500–2500 addresses per km2; 3: moderate 1000–1500 addresses per km2; 4: low 500–1000 addresses per km2; 5: rural <500 addresses per km2. cEnergy from carbohydrates, protein and fat, relative to the sum of energy from the three macronutrients. dBMI = Body mass index. eMedian + IQR among alcohol users. One standard drink contains 10 g alcohol. 1 Willet-Schofield. 2Two sided p-value; Mann Whitney U test for continuous characteristics or Pearson Chi-Square for categorical characteristics. (DOCX) [file pone.0225973.s003.docx]

**S2 Table.** **Characteristics stratified by reliability dietary intake.**

| **Characteristics** | **Reliable dietary intake^1^**  **N=1698 (100%)** | | **Unreliable dietary intake^1^**  **N=427 (100%)** | | **P^2^** |
| --- | --- | --- | --- | --- | --- |
| **Demographics** |  |  |  |  |  |
| Age at enrollment (years) | 29 | (27-32) | 30 | (27-33) | <0.001 |
| Ethnicity |  |  |  |  | 0.18 |
| *White, East/West European Ethnicity* | 1661 | (97.8) | 422 | (98.9) |  |
| *Other* | 37 | (2.2) | 5 | (1.2) |  |
| Education^a^ |  |  |  |  | <0.001 |
| *Low* | 105 | (6.3) | 43 | (10.2) |  |
| *Moderate* | 637 | (37.9) | 184 | (43.6) |  |
| *High* | 936 | (55.8) | 195 | (46.2) |  |
| Missing | 20 |  | 5 |  |  |
|  |  |  |  |  |  |
| Urbanization level by category ^b^ |  |  |  |  | <0.001 |
| *1* | 403 | (24.2) | 77 | (18.4) |  |
| *2* | 133 | (8.0) | 69 | (16.5) |  |
| *3* | 118 | (7.1) | 28 | (6.7) |  |
| *4* | 200 | (12.0) | 37 | (8.8) |  |
| *5* | 812 | (48.7) | 208 | (49.6) |  |
| Missing | 32 |  | 8 |  |  |
|  |  |  |  |  |  |
| **Diet** |  |  |  |  |  |
| Energy intake (kcal/day) | 1813 | (1545-2141) | 75 .2 | (43.4-132.4) | <0.001 |
|  |  |  |  |  |  |
| **Lifestyle** |  |  |  |  |  |
| BMI ^d^ (kg/m^2^) | 23.8 | (21.7-26.6) | 24.1 | (21.9-27.3) | 0.29 |
| BMI WHO classification |  |  |  |  | 0.28 |
| *<18.5* | 19 | (1.1) | 6 | (1.4) |  |
| *18.5-<25* | 1019 | (60.0) | 242 | (56.7) |  |
| *25-<30* | 469 | (27.6) | 137 | (32.1) |  |
| *≥ 30* | 191 | (11.3) | 42 | (9.8) |  |
| Alcohol |  |  |  |  |  |
| *User percentage (%)* | 1317 | (77.6) | 149 | (34.9) | <0.001 |
| *Median consumption*^e^(g/day) | 2.7 | (1.4-6.4) | 1.6 | (1.6-2.5) | <0.001 |
| *Missing* | 2 |  | 0 |  |  |
| Smoker | 212 | (12.5) | 83 | (20.8) | <0.001 |
| *Missing* | 3 |  | 28 |  |  |
|  |  |  |  |  |  |
| **Pregnancy** |  |  |  |  |  |
| Maximum time between baseline questionnaire and birth child (in months) | 13.0 | (11.0-16.0) | 32.0 | (23.0-38.0) | <0.001 |
| Sex of the child |  |  |  |  |  |
| *Male* | 849 | (50.0) | 219 | (51.3) | 0.67 |
| Gravidity |  |  |  |  | <0.001 |
| *1* | 692 | (40.8) | 137 | (32.2) |  |
| *2* | 578 | (34.0) | 133 | (31.3) |  |
| *3* | 275 | (16.2) | 93 | (21.9) |  |
| *≥4* | 153 | (9.0) | 62 | (14.6) |  |
| *Missing* | 0 |  | 2 |  |  |
| Parity |  |  |  |  | <0.001 |
| *0* | 813 | (47.9) | 166 | (39.0) |  |
| *1* | 632 | (37.2) | 154 | (36.2) |  |
| *≥2* | 253 | (14.9) | 106 | (24.9) |  |
| *Missing* |  |  | 1 |  |  |
| Birth weight (in grams) | 3570 | (3249-3880) | 3640 | (3330-3980) | 0.005 |
| *Missing* | 4 |  |  |  |  |
| Gestational age (in weeks) | 39 | (39-40) | 40 | (38-40) | 0.36 |
| Apgar-score (after 5 min) |  |  |  |  | 0.06 |
| *<10* | 422 | (24.9) | 86 | (11.1) |  |
| *10* | 1274 | (75.1) | 341` | (79.9) |  |
| *Missing* | 2 |  |  |  |  |
